# Flow and Immersion in Video Games: The Aftermath of a Conceptual Challenge

**DOI:** 10.3389/fpsyg.2018.01682

**Published:** 2018-09-05

**Authors:** Lazaros Michailidis, Emili Balaguer-Ballester, Xun He

**Affiliations:** ^1^Bournemouth University, Centre for Digital Entertainment, Department of Media and Communication, Bournemouth, United Kingdom; ^2^Sony Interactive Entertainment, London, United Kingdom; ^3^Bournemouth University, Department of Computing and Informatics, Bournemouth, United Kingdom; ^4^Bernstein Centre for Computational Neuroscience Heidelberg-Mannheim, Manheim, Germany; ^5^Bournemouth University, Department of Psychology, Bournemouth, United Kingdom

**Keywords:** flow, immersion, presence, engagement, video games

## Abstract

One of the most pleasurable aspects of video games is their ability to induce immersive experiences. However, there appears to be a tentative conceptualization of what an immersive experience is. In this short review, we specifically focus on the terms of flow and immersion, as they are the most widely used and applied definitions in the video game literature, whilst their differences remain disputable. We critically review the concepts separately and proceed with a comparison on their proposed differences. We conclude that immersion and flow do not substantially differ in current studies and that more evidence is needed to justify their separation.

## Introduction

Video games offer highly positive experiences and it has been argued that the experience of flow alone may be responsible for the positive emotions during video game playing (Hoffman and Novak, 1996; Quinn, 2005; Guo et al., 2012; Nah et al., 2014). Literature, however, assimilates multiple terms that emulate flow’s richness of an experience – for instance, immersion. Although immersion presents subtle structural differences from flow, it is believed that they allude to different mental phenomena (Brown and Cairns, 2004; IJsselsteijn et al., 2007; Jennett et al., 2008b; Nacke and Lindley, 2008, 2009; Brockmyer et al., 2009; Qin et al., 2009; Drachen et al., 2010; Teng, 2010; Kiili et al., 2012; Sweetser et al., 2012; Cairns et al., 2014; Denisova et al., 2017; Frochot et al., 2017). The number of theories that exist for flow and immersion are a testimony to the complex nature of the underlying mental state.

The overt similarities between flow and immersion are available when examining popular theories of immersion (e.g., Brown and Cairns, 2004; Ermi and Mäyrä, 2005; Jennett et al., 2008b; Kiili et al., 2012). For example, concentration, loss of time perception, a balance between the player’s skills and the game’s demands, and loss of self-awareness are some of the mutual properties that both flow and immersion exhibit (e.g., Brown and Cairns, 2004). Immersive experiences during video game playing are still predominantly measured with questionnaires (Procci and Bowers, 2011; Lee et al., 2014; Bian et al., 2016). These tools often present variability on the definitional premise upon which they were structured, and thus raise ambiguity over the validity of what they claim to be capturing. This short review examines the main differences that have been outlined for flow and immersion and argues that these states might actually be the same.

## A Comparison Between Flow and Immersion

### Qualifying the Experience

Immersion is perhaps more popular than flow across academics and non-academics alike (Smith, 2016) and has a long history of interpretations (Smith et al., 1998). The debate, however, is not merely about which term is more appropriate than the other (Cairns, 2016), but mainly about the sensory, cognitive, and emotional products of an immersive experience.

Flow theory is often approached from a radical standpoint, wherein all of its nine proposed dimensions must be present for the experience to qualify as flow. These dimensions include balance between the skills of an individual and the activity’s demands; merging of action and awareness; clear goals; immediate and unambiguous feedback; concentration on the task; perceived control over the activity; loss of self-reflection; distorted perception of time; and intrinsic motivation toward an activity (autotelic) (Csikszentmihalyi, 1975, 1990). Cairns et al. (2014) have suggested that flow is an “all-or-nothing” experience, during which the individual either fulfills all the criteria for flow to kick in, or they do not, in which case flow will not come into effect. Others have argued that flow does not need to comply with all the criteria simultaneously (Csikszentmihalyi, 1990, 1998; Quinn, 2005; Chen, 2007; Guo and Poole, 2009; Heo et al., 2010; Swann et al., 2012; Arzate and Ramirez, 2017; Frochot et al., 2017). However, the minimum requirements for an experience to qualify as flow remains unsettled (i.e., the *necessary* vs. the *sufficient* conditions; Swann et al., 2012). For example, Skadberg and Kimmel (2004) found that the criteria with the highest factor loadings for flow were enjoyment and time perception distortion. In contrast, Klasen et al. (2012) found that the conjunction of balance between challenge and skills, sense of control and concentration were the most representative of the flow experience.

This possible limitation in flow theory has been exploited in favor of new definitions for an immersive experience. However, it should be noted that Csikszentmihalyi’s (1975, 1990) list of flow criteria relied on oral report patterns between multiple interviews. In 1990 (p. 49) he mentioned, “*When people reflect on how it feels when their experience is most positive, they mention at least one, and often all, of the following. (…).*” Thus, flow’s criteria were compiled from a list of the most commonly reported sensations during flow and might not be universally applicable to every person or every instance of an activity (e.g., Nakamura and Csikszentmihalyi, 2014). We argue that flow’s dimensions are more descriptive in nature rather than definitive. Similarly, the characteristics of each stage in immersion describe the average immersive episode, but they are not guaranteed (Brown and Cairns, 2004).

### Experiential Extremity

Flow has been referred to as the *optimal* experience when *nothing else matters* (Csikszentmihalyi, 1990; Jackson and Csikszentmihalyi, 1999). This characterization has led to the belief that flow is a particularly intense experience, and therefore extreme (Jennett et al., 2008b; Sanders and Cairns, 2010; Frochot et al., 2017), which makes immersion an antecedent to flow (Jennett et al., 2008b; Seah and Cairns, 2008; Brockmyer et al., 2009; Nacke and Lindley, 2009; Sanders and Cairns, 2010). Importantly, proponents of this notion have yet to address how immersion can further descend into flow and to trace the qualifying elements for such a transition.

Immersion, on the other hand, has been tagged as *sub-optimal*, a characterization that has been deemed to be more suitable for video game playing (Jennett et al., 2008b; Cheng et al., 2015). Brown and Cairns (2004) identified three grades of immersion: engagement, engrossment and total immersion, and argued that total immersion is not always achievable (see also, Jennett et al., 2008a). Hence, the model of an average immersive experience in video game playing can be reduced to the engagement and engrossment levels, whose characteristics are not considerably divergent from flow. Although immersion is purported to be a non-extreme state, Brown and Cairns put forward the concept of total immersion, whose name also denotes an experiential extremity. This appears to be the equivalent of Csikszentmihalyi (1992)’s micro-flow and deep-flow episodes – in this sense, total immersion would qualify as a deep-flow episode, which occurs more rarely than micro-flow episodes do.

Given the similarities between flow and immersion, it is not safe to conclude that flow is more “extreme” than immersion. The mechanisms, indicative of an extreme experience, are likely to be concentration, loss of self-reflection, distortion of time perception and autotelicity, all of which make flow inherently dissociative from reality. Except for autotelicity, the remaining dimensions are also found in immersion (e.g., Brown and Cairns, 2004; Jennett et al., 2008b).

To address the last, most distinctive concomitant of the flow experience, autotelicity, we should note that the term poses a conflict in itself. On the one hand, it is used to describe the intrinsic motivation toward an activity, which is evident from the blend word “auto” and “telos”, i.e., to perform the activity is a goal in itself (Csikszentmihalyi, 1990). This meaning renders autotelicity antecedent to flow. On the other hand, autotelicity has been used to denote high satisfaction derived from an activity (Csikszentmihalyi, 1990) – perhaps a product of time perception distortion (e.g., Conti, 2001; Rau et al., 2006; Wood et al., 2007; Luthman et al., 2009; Sackett et al., 2010). Hence, it is treated as an outcome of flow (e.g., Weibel and Wissmath, 2011; Guo et al., 2012; Nah et al., 2014).

Csikszentmihalyi (1990, 1996) mentioned that a person might not always receive satisfaction whilst engaging in an activity, but rather immediately after. In video games, it has been argued that positive and negative emotions are both valid constituents of an engaging experience (Kaye et al., 2018; Silpasuwanchai and Ren, 2018). These observations indicate that autotelicity might be asynchronous to the core flow experience (Quinn, 2005; Engeser and Schiepe-Tiska, 2012), and conflict with the notion that flow is more extreme than immersion. Toward a more precise identification of experiential intensity, it is critical to quantify an immersive episode by its duration, latency (time taken to trigger the episode), intensity, and frequency of breaks (how often the episodes are interrupted).

### The Interplay Between Flow, Immersion, and Presence

Among the constructs that have been traditionally associated with video game playing, “presence” often appears in tandem with both flow and immersion (e.g., McMahan, 2003; Nah et al., 2014). For clarity, this article uses presence in lieu of “spatial presence” (for reviews, see Tamborini and Skalski, 2006; Sjölie, 2014). Presence is elicited when the player feels as being in the game (e.g., Brown and Cairns, 2004; Brockmyer et al., 2009) – it is considered a highly relevant concept for video game playing (Tamborini and Skalski, 2006), but its conceptualization is often confounded with that of immersion (McMahan, 2003).

Immersion theories have incorporated presence to an extent that it becomes indistinguishable from immersion (e.g., McMahan, 2003; Brown and Cairns, 2004; Calleja, 2007; Norman, 2010; Kiili et al., 2012). For example, Brown and Cairns (2004) have explicitly equated total immersion to presence. Their framework visualizes immersion as a multi-graded construct, that intensifies over time, yet presence fails to provide the quantitative information needed to separate it from earlier, or less intense, stages of immersion. Moreover, the authors inferred that engagement and engrossment – which encompass physical and emotional investment in the game, loss of self-awareness and sustained attention – *prime* the experience of presence. Consequently, the model describes the path to attaining presence, which is viewed as the core of immersion, even if players do not always progress into presence (Jennett et al., 2008a; Skalski et al., 2011). Thus, we suggest that the model of Brown and Cairns possibly yields an incomplete taxonomy.

In contrast, the distinction between presence and flow is perhaps clearer. According to Weibel and Wissmath (2011), flow is the sensation of influencing the activity in the virtual world (“gaming action”), whereas presence is the sense of being in the virtual world. This distinction exposes a fundamental difference: presence may not necessitate player interaction or physical effort (Slater and Wilbur, 1997; IJsselsteijn et al., 2000; Nicovich et al., 2005; Baumgartner et al., 2006). Essentially, presence is fostered by a feed-forward loop that seeks to match the user’s mental representations of the real world with the virtual world (Schuemie et al., 2001; Riva et al., 2003; Jäncke et al., 2009; Sjölie, 2014). With the addition of presence, immersion becomes an over-inclusive concept, thereby fueling the differentiation between immersion and flow (e.g., Calleja, 2007; Nacke and Lindley, 2008; Kiili et al., 2012).

However, presence and flow also present similarities. Witmer and Singer (1998) suggested that the prerequisites for presence are concentration, a sense of control, and the presence of feedback, which are some of the same criteria Csikszentmihalyi (1975, 1990) suggested for flow. In addition, both presence and flow have been associated with decreased frontal brain activation (e.g., Dietrich, 2004; Jäncke et al., 2009; Clemente et al., 2013). Finally, the maintenance of these states is achieved through selective attention (Witmer and Singer, 1998; Kober and Neuper, 2012; Harris et al., 2017a).

It is possible that presence operates separately from flow, and that these two states share similar pre-conditions (Mollen and Wilson, 2010). However, it is also possible that it is experienced *before* flow (e.g., Novak et al., 2000; Skadberg and Kimmel, 2004; Weibel et al., 2008; Weibel and Wissmath, 2011). To conclude, presence appears to refer to a different sensation from the experience of immersion or flow. Arguably, flow and immersion present the characteristics of an altered state of consciousness, whereas presence does not (Brockmyer et al., 2009). This distinction places presence at an early stage of video game engagement (e.g., Weibel and Wissmath, 2011; Klasen et al., 2012).

### Neural Correlates Underlying Flow, Immersion, and Presence

Although research seems to lack robust evidence for neural *patterns* observable in flow, immersion, and presence, these constructs may be sharing mutual neural correlates (Klasen et al., 2012). However, this absence of patterns, specific to each construct, may be due to the particularities of the tasks employed in neural studies.

Flow has been associated with the dopaminergic system (Marr, 2001; Weber et al., 2009; De Manzano et al., 2013; Gyurkovics et al., 2016), the sensorimotor network (Sanchez-Vives and Slater, 2005; Klasen et al., 2012), the combination of anterior cingulate cortex and temporal pole (Yun et al., 2017), and reduced activity in the prefrontal cortex (Gusnard et al., 2001; Dietrich, 2004; Goldberg et al., 2006; Bavelier et al., 2012; Ulrich et al., 2014). The prefrontal cortex (PFC) remains a debated brain structure for its role in flow. Dietrich (2004) hypothesized that flow reflects reduced frontal brain activity, and basal ganglia are deployed to allow a state of automatic behavior and executive functioning – a state known as transient hypofrontality (Dietrich, 2004).

The state of hypofrontality has not been consistently confirmed (Yoshida et al., 2014; Harmat et al., 2015; Harris et al., 2017b). In a recent study by Causse et al. (2017), the authors found that the PFC activity is proportional to the task’s demands. However, explicit control on the task may not be essential during flow (e.g., Taylor, 2002; Dietrich, 2004; Bavelier et al., 2012). Indeed, task automation is a distinctive feature of flow (e.g., Csikszentmihalyi, 1975; Quinn, 2005), and it progressively loses its dependency on the PFC as expertise increases (Léger et al., 2014). Klasen et al. (2012) recruited expert players and a conjunction analysis of flow factors revealed a neocerebellar-somatosensory network. Contrarily, Yoshida et al. (2014) found increased activity in the left and right ventrolateral PFC during flow. However, Yoshida et al. did not mention whether the participants in their study were skilled players. This is an important factor, in that flow may orchestrate neural networks differently for novice and expert players (e.g., Kirschner and Williams, 2014), with novice players requiring more explicit control (e.g., Dietrich, 2004).

Similarly, evidence on the loss of self-reflective thoughts during flow has revealed reduction in the activity of the medial PFC (Ulrich et al., 2014), whereas top-down attention implicated in flow (Harris et al., 2017a) has been associated with the lateral PFC and frontal eye fields (Buschman and Miller, 2007). These contradicting findings should be interpreted with care; flow is perhaps related to a localized hypofrontality, and not as universal as Dietrich (2004) originally speculated (Harris et al., 2017b). We suggest that frontal brain reduction may be a function of time. For example, Yun et al. (2017) found that flow peaked after 25 min of game playing. Hence, the player may require time to transition from explicit to implicit control and to allocate attentional resources that will make him/her resistant to distractions (e.g., Jackson and Csikszentmihalyi, 1999; Jennett, 2010; Bavelier et al., 2012; Nuñez Castellar et al., 2016). Although similar observations have not been proposed for immersion, the conceptual overlap between flow and immersion (Brown and Cairns, 2004; Jennett et al., 2008b) suggests that mutual substrates are at play.

Klasen et al. (2012) have additionally identified the role of parietal regions (i.e., superior parietal cortex, precuneus and intraparietal sulcus) in flow. However, an occipital and frontoparietal network has also been found to underlie spatial awareness in the first-person perspective (Vogeley and Fink, 2003; Vogeley et al., 2004) – Klasen et al. used a first-person video game in their study. This finding is alarming in that concurrent neural networks, specific to the task, may have been misidentified for flow dimensions, as argued by Yoshida et al. (2014). Different game genres can have specific cognitive demands (e.g., Latham et al., 2013), and, as such, certain video games (e.g., strategy games) might require more explicit control than others (e.g., Spence and Feng, 2010). These games might stimulate higher prefrontal activation, without necessarily implying that it is a substrate of flow. Importantly, the frontoparietal network’s contribution to the experience of flow remains questionable (Bavelier et al., 2012; Léger et al., 2014; Nah et al., 2017). The responsibility of the frontoparietal network to allocate attentional resources and the reduced activation thereof in video game playing (Bavelier et al., 2012) suggests a functional unrelatedness to flow. This is evident from flow’s dimension “merging of action and awareness”, which signifies that attention is already focused (Csikszentmihalyi, 1975).

On the other hand, presence has received limited focus on its neural substrates. From the few studies investigating them, presence has been consistently shown to rely on frontoparietal connections (e.g., Baumgartner et al., 2006, 2008; Jäncke et al., 2009; Kober et al., 2012; Clemente et al., 2013). Jäncke et al. (2009) found that the dorsolateral PFC not only does it regulate the sense of presence but also its activation is inversely related to presence. The authors suggested that the role of the frontoparietal network is essential to presence because it governs motor simulations extracted from the user’s internal representations (see also, Sjölie, 2014). Although motor simulation and execution have been shown to have a strong overlap on a neural level (Hesslow, 2002), research has shown distinct boundaries.

For instance, Ingvar and Philipson (1977) found that motor simulation resided in a frontoparietal network, whereas motor execution was associated with the Rolandic area. Similarly, Bauer et al. (2015) found that a network, including the left parietal, motor areas and the right PFC, underlay motor simulation, whereas motor execution was related to left and right motor areas. These findings are consistent with the alleged relation of presence to action representation, or the ability to “do there” (e.g., Sanchez-Vives and Slater, 2005; Baumgartner et al., 2008; Sjölie, 2014).

Presence has also been referred to as an “out-of-body” experience (Rheingold, 1991), which is the equivalent of “being there” (Sanchez-Vives and Slater, 2005). The user becomes dissociated from the surrounding environment and feels like they lose ownership of their body (Sanchez-Vives and Slater, 2005). Out-of-body experiences have been consistently shown to involve the temporoparietal junction (Blanke and Arzy, 2005; Bünning and Blanke, 2005; Blanke et al., 2005; De Ridder et al., 2007), which gives rise to illusory self-location and perspective (Blanke and Arzy, 2005). As such, presence has been argued to be resembling to an out-of-body experience (Herbelin et al., 2016) and links to the temporoparietal junction have been found in studies of presence (Baumgartner et al., 2006, 2008).

These findings may not offer direct evidence for a comparison between flow, immersion and presence. However, they shed light on some differences. Flow is triggered during a task, thereby implying motor execution. Contrarily, presence may be more related to motor simulation (Sjölie, 2014). As mentioned above, motor execution and simulation share functional correlates, but they also present differences. This notion supports Weibel and Wissmath (2011)’s insights on the distinction between flow and presence. Moreover, presence appears to be a visceral sensation and a primal mechanism of sensory integration (e.g., Riva and Waterworth, 2003), whereas flow and immersion require increased mental effort in a task (e.g., Csikszentmihalyi, 1990; Brown and Cairns, 2004; Keller et al., 2011; Weibel and Wissmath, 2011), thus functionally evolving over time (e.g., De Lafuente and Romo, 2006). In addition, the frontoparietal network appears to be essential for the sense of presence (Kober et al., 2012) but not for flow (Bavelier et al., 2012; Léger et al., 2014; Nah et al., 2017).

Presence is challenging to isolate in video games, because interactivity is their key component when compared to other media forms (Granic et al., 2014). Perhaps, this very challenge has sparked the unification between immersion and presence (e.g., McMahan, 2003; Brown and Cairns, 2004; Calleja, 2007; Kiili et al., 2012). Contrary to the growing body of literature investigating the neural correlates of flow (e.g., Harris et al., 2017b; Nah et al., 2017; Tian et al., 2017), there is, in our view, a gap in the mechanisms underlying immersion, rendering their distinction a conceptual challenge.

## Concluding Remarks

This focused review briefly addressed some conceptual challenges in the literature of flow vs. immersion and their related concept of presence in video gaming. Our aim was to challenge the dominant view that flow is different from immersion. Although the theoretical debate may seem innocuous, it extends to experimental settings of substantial variability. Currently, there is lacking evidence to suggest that a particular game design is better at triggering flow than immersion or vice versa (see, Nacke and Lindley, 2008, 2009; Nacke et al., 2010).

Despite the abundance and overlap of self-reports (Nordin et al., 2014; Denisova et al., 2016), research has not been effective in diversifying the approaches toward the measurement of flow and immersion as separate states. Importantly, existing and future studies, guided by opposing interpretations of the same phenomenon, may result in generalizability issues.

To conclude, immersion and flow do not appear as conceptually distinct, and their proposed differences are not compelling enough to set immersion apart as a different mental state. Although presence is enveloped in immersion, it appears to be a distinct mental state, even on a neural level. The remaining dimensions of immersion are very similar, if not identical, to flow’s. Thus, we suggest that the terms of flow and immersion can be used interchangeably, until further behavioral and neurophysiological evidence is provided in experimental settings specifically designed for disentangling the two states.

## Author Contributions

All authors listed have made a substant ial, direct and intellectual contribution to the work, and approved it for publication.

## Conflict of Interest Statement

The authors declare that the research was conducted in the absence of any commercial or financial relationships that could be construed as a potential conflict of interest.
